# Some bacteria degrade explosives, others prefer boiling methanol

**DOI:** 10.1111/j.1462-2920.2007.01480.x

**Published:** 2007-12-01

**Authors:** Michael Y Galperin

**Affiliations:** National Center for Biotechnology Information, National Library of Medicine, National Institutes of Health Bethesda, Maryland 20894, USA

The list of completely sequenced microbial genomes, released in August and September of 2007 (Table 1), is relatively short. Still, it includes some remarkable environmental microorganisms, such as the sulfur-reducing crenarchaeon *Ignicoccus hospitalis*, host of the smallest archaeon *Nanoarchaeum equitans*, the soil bacterium *Bacillus pumilus* isolated from a supposedly sterile environment of the spacecraft assembly facility in Pasadena, California, a marine bacterium that degrades nitramine explosives and two enterobacteria that are commonly found in soil and water habitats but can also infect humans, particularly newborns, causing sepsis and neonatal meningitis.

**Table 1:** Recently completed microbial genomes (August–September 2007).

Species name	Taxonomy	GenBank accession	Genome size, bp	Proteins (total)	Sequencing centre^a^	Reference
**New organisms**						
*Giardia lamblia*	*Eukaryota, Diplomonadida*	AACB02000000	11 700 000	6470	MBL	Morrison *et al.* (2007)
*Ignicoccus hospitalis*	*Crenarchaeota*	CP000816	1 297 538	1434	JGI	Unpublished
*Roseiflexus castenholzii*	*Chloroflexi*	CP000804	5 723 298	4330	JGI	Unpublished
*Bacillus pumilus*	*Firmicutes*	CP000813	3 704 465	3681	Baylor	Gioia *et al.* (2007)
*Streptococcus gordonii*	*Firmicutes*	CP000725	2 196 662	2051	JCVI	Unpublished
*Rickettsia akari*	*α-Proteobacteria*	CP000847	1 231 060	1259	U. Iowa	Unpublished
*Rickettsia canadensis*	*α-Proteobacteria*	CP000409	1 159 772	1093	U. Iowa	Eremeeva *et al.* (2005)
*Rickettsia massiliae*	*α-Proteobacteria*	CP000683	1 360 898	980	CNRS-Marseille	Blanc *et al.* (2007a)
		CP000684	15 286			
*Rickettsia rickettsii*	*α-Proteobacteria*	CP000848	1 257 710	1345	U. Iowa	Unpublished
*Citrobacter koseri*	*γ-Proteobacteria*	CP000822	4 720 462	5031	WashU	Unpublished
		CP000823	9 294			
		CP000824	5 601			
*Enterobacter sakazakii*	*γ-Proteobacteria*	CP000783	4 368 373	4442	WashU	Unpublished
		CP000785	131 196			
		CP000784	31 208			
*Serratia proteamaculans*	*γ-Proteobacteria*	CP000826	5 448 853	4942	JGI	Unpublished
		CP000827	46 804			
*Shewanella pealeana*	*γ-Proteobacteria*	CP000851	5 174 581	4241	JGI	Unpublished
*Shewanella sediminis*	*γ-Proteobacteria*	CP000821	5 517 674	4497	JGI	Unpublished
*Vibrio harveyi*	*γ-Proteobacteria*	CP000789	3 765 351	6064	WashU	Unpublished
		CP000790	2 204 018			
		CP000791	89 008			
*Arcobacter butzleri*	*ε-Proteobacteria*	CP000361	2 341 251	2259	USDA-ARS	Kaakoush *et al.* (2007)
*Campylobacter concisus*	*ε-Proteobacteria*	CP000792	2 052 007	1985	JCVI	Unpublished
		CP000793	30 949			
		CP000794	16 457			
*Thermotoga lettingae*	*Thermotogae*	CP000812	2 135 342	2040	JGI	Unpublished
**New strains**						
*Prochlorococcus marinus* str. MIT 9215	*Cyanobacteria*	CP000825	1 738 790	1983	JGI	Unpublished
*Staphylococcus aureus* subsp. *aureus* Mu3	*Firmicutes*	AP009324	2 880 168	2698	Juntendo U.	Unpublished
*Rickettsia bellii* OSU 85-389	*α-Proteobacteria*	CP000849	1 528 980	1476	U. Iowa	Unpublished
*Escherichia coli* E24377A	*γ-Proteobacteria*	CP000795-CP000801	5 249 288(total)	4997	JCVI	Unpublished
*Escherichia coli*HS	*γ-Proteobacteria*	CP000802	4 643 538	4384	JCVI	Unpublished
*Francisella tularensis* subsp. *holarctica* FTA	*γ-Proteobacteria*	CP000803	1 890 909	2079	JGI	Unpublished
*Campylobacter jejuni* subsp. *jejuni* 81116	*ε-Proteobacteria*	CP000814	1 628 115	1626	IFR-Norwich	Pearson *et al.* (2007)

a Sequencing centre names are abbreviated as follows: Baylor, Human Genome Sequencing Center, Baylor College of Medicine, Houston, Texas, USA; CNRS-Marseille, CNRS-UPR 2589, Institut de Biologie Structurale et Microbiologie, Marseille, France; IFR-Norwich, Institute of Food Research, Norwich Research Park, Norwich, U.K.; JCVI, J. Craig Venter Institute, Rockville, Maryland, USA; JGI, US Department of Energy Joint Genome Institute, Walnut Creek, California, USA; Juntendo U., Department of Bacteriology at Juntendo University, Bunkyo-ku, Tokyo, Japan; MBL, Marine Biological Laboratory, Woods Hole, Massachusetts, USA; U. Iowa, Environmental Health Sciences Research Center, The University of Iowa, Iowa City, Iowa, USA; USDA-ARS, US Department of Agriculture, Agricultural Research Service, Western Regional Research Center, Albany, California, USA; WashU, Washington University School of Medicine, St. Louis, Missouri, USA.

The largest of the recently sequenced genomes comes from the early-diverging amitochondrial eukaryote *Giardia lamblia* (Morrison *et al.*, 2007). Like most other eukaryotic genomes, it has been released in a draft form that consists of 306 contigs, representing 5 chromosomes of *G. lamblia*. In accordance with earlier reports, *G. lamblia* encodes a simplified archaeal-like DNA replication machinery, a yeast-like machinery for transcription synthesis and RNA processing and a limited set of largely bacterial-like metabolic enzymes. Since *G. lamblia* is an intestinal parasite, its primitive features could be equally well rationalized as either an ancestral state of the eukaryotic cell or as a result of a later adaptation to the parasitic lifestyle. So, although the genome of *G. lamblia* is certainly an important step towards understanding the origin and early evolution of the eukaryotic cell, genomes of other early-diverging eukaryotes would be needed to allow meaningful comparative analysis.

*Ignicoccus hospitalis,* isolated from a submarine hydrothermal vent to the north of Iceland and originally described as *Ignicoccus*sp. KIN4/I, is an interesting organism in its own right. It is an obligately anaerobic hyperthermophilic chemolitoautotroph that uses CO_2_ as a source of carbon and derives energy from reducing elemental sulfur with H_2_ as the sole electron donor (Paper *et al.*, 2007). It belongs to a recently described genus that forms a deeply branching lineage within the crenarchaeal family *Desulfurococcaceae* (Huber *et al.*, 2000) and has an outer membrane (Näther and Rachel, 2004) and an unusual pathway of autotrophic CO_2_ fixation (Jahn *et al.*, 2007). Still, *Ignicoccus* never attracted as much attention as the tiny (∼400 nm) coccoidal cells of *Nanoarchaeum equitans* found on its surface. Based on its unique 16S rRNA sequence and the extremely small size of its genome (less than 0.5 Mbp), *N. equitans* was assigned to a separate phylum of archaea, the *Nanoarchaeota* (Huber *et al.*, 2002). Subsequent genome sequencing revealed an extremely reduced genome of only 491 kb with 536 protein-coding genes (Waters *et al.*, 2003). These genes encoded the components of information processing and DNA repair machinery, but not enzymes of lipid, cofactor, amino acid, or nucleotide biosynthesis. These observations showed that *N. equitans* must acquire most of its biosynthetic precursors from its host and cannot exist without it, establishing its interaction with *I. hospitalis* as a kind of symbiotic or parasitic relationship. Although the lack of the core metabolic genes suggested that *N. equitans* was a highly evolved organism, adapted to the parasitic lifestyle, an analysis of its ribosomal genes supported its deep branching at the base of the archaeal phylogenetic tree (Huber *et al.*, 2003; Waters *et al.*, 2003). Subsequent analysis led some researchers to position *N. equitans* near the root of the universal Tree of Life (Di Giulio, 2007), while others argued that it simply belongs to a fast-evolving lineage within the *Euryarchaeota* (Brochier *et al.*, 2005; Makarova and Koonin, 2005). The genome of *I. hospitalis* is expected to shed light on the mechanisms and evolutionary history of its association with *N. equitans*. In addition, it should show whether *I. hospitalis* acquired any of its metabolic genes through lateral gene transfer from *N. equitans.* However, for a better resolution of ancestral archaeal phylogeny, we should probably wait for the upcoming release by the JGI of the genome of *Korarchaeota* OPF1-KOR, a representative of yet another ancient lineage, *Korarchaeota*, which is currently under embargo (see http://genome.jgi-psf.org/mic_cur1.html).

*Roseiflexus castenholzii* is a facultatively anaerobic moderately thermophilic filamentous phototrophic bacterium that belongs to the phylum *Chloroflexi*, also known as *green non-sulfur bacteria*, which unifies filamentous bacteria that lack peptidoglycan in their cell walls (Meissner *et al.*, 1988). This is the 5^th^ completely sequenced genome from that phylum, coming on the heels of the genome of *Roseiflexus* sp. strain RS-1 that has been released by the JGI earlier this year and three genomes of *Dehalococcoides* spp. The genome of *Chloroflexus aurantiacus*, the best-studied representative of the *Chloroflexi*, is available in GenBank (accession no. AAAH00000000) in an unfinished form. The sequenced strain *Roseiflexus castenholzii* DSM 13941 has been isolated from a red-colored bacterial mat developed in Nakabusa hot spring near Nagano, Japan (Hanada *et al.*, 2002). Although similar to *C. aurantiacus* in manyfeatures, including the cell shape, gliding motility and the ability to perform anoxygenic photosynthesis, *R. castenholzii* does not contain chlorosomes or bacteriochlorophyll *c*; its major photosynthetic pigment is bacteriochlorophyll *a. Roseiflexus castenholzii* also differs from other members of the family *Chloroflexaceae* in that its cells are actually red, owing to the high amount of γ-carotene (Hanada *et al.*, 2002). The organization of genes encoding the photosynthetic reaction center and the light-harvesting proteins of *R. castenholzii* differs from that in *C. aurantiacus* (Yamada *et al.*, 2005), which could help in understanding the evolution of anoxygenic photosynthesis and photosynthesis in general.

The next organism in the list (Table 1), *Bacillus pumilus*, is a common Gram-positive soil bacterium that is often associated with plant roots and has been studied primarily because of its role in plant defense against fungal and nematode parasites. *Bacillus pumilus* is often found in various foods and on some occasions has been identified as a source of food poisoning. The ability of *B. pumilis* to survive standard sterilization procedures has been attributed the high resistance of its spores to gamma irradiation and common solvents. These properties have become subject of intense interest in 1999–2002 when spores of *B. pumilis* and several other bacilli were isolated from the spacecraft surfaces at the Spacecraft Assembly Facility of the NASA Jet Propulsion Laboratory in Pasadena, California (Kempf *et al.*, 2005). One of the strains, *B. pumilus* SAFR-032, isolated from a clean-room airlock, demonstrated unusually high resistance to UV radiation and was even able to withstand UV irradiation in the 200 to 400 nm range at the levels that were expected to be found on the surface of Mars (Newcombe *et al.*, 2005). Although direct exposure to extremely short-wavelength (10–100 nm) UV irradiation that exists in high vacuum appears to effectively kill both spores and vegetative cells (Saffary *et al.*, 2002), these data revived the idea that such spores could survive space travel under the surface of basalt rocks and be brought to Earth from other planets (Benardini *et al.*, 2003). The complete genome of *B. pumilus* strain SAFR-032 has now been sequenced and its adaptations to UV irradiation and peroxide stress analyzed in detail (Gioia *et al.*, 2007). Surprisingly, *B. pumilus* SAFR-032 encoded essentially the same set of genes related to DNA repair and H_2_O_2_ resistance as far less UV-resistant *B. subtilis* and *B. licheniformis* (Gioia *et al.*, 2007). Still, certain differences in gene order and deduced protein sequences were identified and will be subject of further analysis. This observation is somewhat similar to the results of the just-published comparative analysis of the complete genomes of *Deinococcus radiodurans* and *Deinococcus geothermalis* (Makarova *et al.*, 2007), which did not reveal any specific genes responsible for the extreme radioresistance of these two organisms. Anyway, whether or not *B. pumilus* could have come from other planets – or whether or not our space probes could contaminate the Martian atmosphere along the lines outlined by Ray Bradbury – the extreme resistance of this organism to sterilization protocols deserves a careful consideration.

The second Gram-positive bacterium on the list, *Streptococcus gordonii*, is a normal inhabitant of human oral cavity. It has been implicated in the development of dental caries and gum disease. From the oral cavity, *S. gordonii* can spread to the bloodstream, causing bacterial endocarditis. The sequenced strain *S. gordonii* Challis is commonly used as a model organism and is relatively well studied.

The past two months brought 5 new genomes of *Rickettsia* spp. (Table 1), doubling the total number of complete rickettsial genomes. This genus, named after Howard Taylor Ricketts (1871–1910), who described the first bacterium of this group exactly 100 years ago, consists of arthropod-borne *α-Proteobacteria*, some of which are important pathogens. Human diseases caused by rickettsiae include epidemic typhus (caused primarily by *Rickettsia prowazekii* and *Rickettsia typhi)*, scrub typhus (*Rickettsia tsutsugamushi*), the Rocky Mountain spotted fever (*Rickettsia rickettsii*), Mediterranean spotted fever (*Rickettsia conorii*), and rickettsialpox (*Rickettsia akari*). Rickettsiae are obligately intracellular pathogens that are transmitted to their vertebrate hosts by ticks, mites or lice. In 2005 and 2006, *Annals of the New York Academy of Sciences* dedicated two special volumes to the anniversary of the Ricketts' discovery and the current research of rickettsiae (Hechemy *et al.*, 2005, 2006). Analysis of rickettsial genomes has been used to uncover the principles of their evolution (Blanc *et al.*, 2007b) and their relation to the mitochondria (Andersson *et al.*, 1998). Now, analysis of the *Rickettsia massiliae* genome revealed a mobile genetic element containing *tra* gene cluster, shared with *Rickettsia bellii* (Blanc *et al.*, 2007a). This work suggests that lateral gene transfer could have played an important role in the evolution of obligate intracellular bacteria.

The list of the recently sequenced γ-proteobacterial genomes includes 5 representatives of the family *Enterobacteriaceae*, as well as two marine bacteria, and a new strain of tularemia-causing *Francisella tularensis* (Table 1). Two of these, *Citrobacter koseri* (formerly *Citrobacter diversus*) and *Enterobacter sakazakii*, are common environmental organisms, found in soil, water and sewage samples. Although these organisms are often isolated from human feces, they are usually considered to be part of normal gut flora. However, they can turn into dangerous pathogens, particularly for infants. Thus, *C. koseri* is an opportunistic pathogen of the central nervous system that causes sepsis and meningitis in newborn children and in immuno-compromised adults (Doran, 1999). The sequenced strain *C. koseri* ATCC BAA-895 was isolated in 1983 from a case of neonatal meningitis. Similarly, *E. sakazakii* has been repeatedly isolated from infant formula, milk powder, cereals and other sources, and implicated in a number of foodborne diseases causing severe meningitis or enteritis (Nazarowec-White and Farber, 1997; Drudy *et al.*, 2006). The sequenced strain *E. sakazakii* ATCC BAA-894 was isolated from the cerebrospinal fluid in the case of fatal neonatal meningitis in an infant fed with a commercial powdered milk formula in Tennessee in 2001.

The third sequenced member of the *Enterobacteriaceae*, *Serratia proteamaculans*, is a common plant endophyte, isolated from the roots of the poplar tree (Moore *et al.*, 2006). This organism appears to promote plant growth, although the nature of the specific compounds involved in this process remains unknown. The availability of *S. proteamaculans* genome sequence should give a boost to the studies of bacteria-to-plant signals.

The two newly sequenced strains of *Escherichia coli* represent the extreme diversity of this species. *Escherichia coli* strain E24377A (serotype O139:H28) is an enterotoxigenic strain, capable of causing traveler's diarrhea, a nasty disease familiar to most international travelers. This strain produces two types of pili, used for colonization of the surface of small intestine, as well as heat-stable and heat-labile enterotoxins that are largely responsible for its virulence. Genome sequencing reveals a 5-Mb chromosome and 6 plasmids, ranging in size from 5 to 79 kb. These plasmids carry a number of uncharacterized genes, at least some of which might be involved in virulence. In contrast, *Escherichia coli* strain HS appears to be able to colonize the human gastrointestinal tract without causing any obvious disease and is a good model organism to study the colonization mechanisms.

*Shewanella sediminis* strain HAW-EB3 is a recently described marine γ-proteobacterium with potential use in bioremediation (Zhao *et al.*, 2005). It has been isolated from the sediment of Emerald Basin, a former military dumping site of unexploded ordnance located in the Atlantic Ocean, 50 miles from the Halifax Harbor in Nova Scotia, Canada, at the depth of 215 m. This site is heavily contaminated with hexahydro-1,3,5-trinitro-1,3,5-triazine, which is used as an explosive agent and also as a rodenticide and is known under the trade names RDX, hexogen, hexolite, and cyclonite (see http://pubchem.ncbi.nlm.nih.gov/summary/summary.cgi?cid=8490 for the chemical formula and references). The isolated strain *S. sediminis* HAW-EB3 was able to degrade hexogen anaerobically at 10°C, suggesting that it could be used in remediation of various nitramine compounds (Zhao *et al.*, 2004). Given that other *Shewanella* spp. reduce Cr(VI), U(VI), and other toxic metals, these organisms demonstrate a very impressive ability to clean up after humans.

Another new species of *Shewanella* isolated from the Emerald Basin, *Shewanella halifaxensis* (Zhao *et al.*, 2006), turned out to be a relative of the squid symbiont *Shewanella pealeana*, which prompted sequencing of that genome as well. *Shewanella pealeana* inhabits accessory nidamental gland, an oval secretory organ in the female reproductive system of the squid *Loligo pealei* (Leonardo *et al.*, 1999). This gland is remarkable for turning from colorless to red-orange during sexual maturation of the squid, most likely owing to accumulation of carotenoid pigments in the bacterial community inhabiting it (Barbieri *et al.*, 2001). Although the exact function of accessory nidamental gland is unknown, it is believed to participate in the formation of egg capsular sheath, which contains a dense culture of bacteria that may protect cephalopod eggs from predators. *Shewanella pealeana* is a facultatively anaerobic, psychrotolerant bacterium that can grow anaerobically on lactate using elemental sulfur, iron, manganese, nitrate, fumarate, trimethylamine-N-oxide, or thiosulfate as electron acceptors (Leonardo *et al.*, 1999). Comparison of this genome with 13 other completely sequenced genomes of *Shewanella* spp. should provide interesting clues into coevolution of *S. pealeana* and squid cells.

*Vibrio harveyi* is a widespread marine bacterium, often found associated with marine animals, such as octopi and shrimp. For a number of years, it has been a favorite model organism to study regulation of bioluminescence by quorum sensing (Dunlap, 1999). It has a very complex regulatory system (Waters and Bassler, 2006) that should now become much easier to comprehend.

The three new ε-proteobacterial genomes in the current list all come from the family *Campylobacteriaceae*. *Arcobacter butzleri* is an aerotolerant waterborne campylobacterium that is commonly found in pigs, cattle, sheep, and poultry, as well as in surface, drinking, and well water and in processed meat (Snelling *et al.*, 2006). Consumption of contaminated water or infected poultry may lead to human infection. The sequenced strain *A. butzleri* RM4018 was isolated from a case of human gastroenteritis. Meanwhile, the *Campylobacter* sequencing project at the JCVI released the genome of the gastrointestinal clinical isolate *Campylobacter concisus*, the first genome from that species, whereas the Institute of Food Research in Norwich, UK, sequenced the genome of the commonly used laboratory strain *Campylobacter jejuni* subsp. *jejuni* 81116, which is infective for chicken (Pearson *et al.*, 2007). This brings to 17 the total number of completely sequenced genomes of representatives of the families *Campylobacteriaceae* and *Helicobacteriaceae*, including genomes of two free-living bacteria, *Thiomicrospira denitrificans* and *Wolinella succinogenes*. Given the recent progress in sequencing the genomes of free-living ε-proteobacteria of unclear phylogenetic status that inhabit deep-sea thermal vents (Nakagawa *et al.*, 2005; 2007), this class of *Proteobacteria* is finally achieving reasonable genome coverage.

The *Thermotogales* sequencing project at the JGI released the complete genome of yet another representative of this early-branching bacterial phylum. *Thermotoga lettingae* strain TMO was isolated in 2002 from a sulfate-reducing bioreactor operated at 65°C with methanol as the sole carbon and energy source (Balk *et al.*, 2002). *Thermotoga lettingae* fermented methanol to acetate, CO_2_ and H_2_. In the presence of electron acceptors, such as thiosulfate, elemental sulfur, or Fe(III), it was able to degrade methanol to CO_2_ and H_2_ (Balk *et al.*, 2002). The unique ability of *T. lettingae* to utilize methanol near its boiling point of 64.7°C makes it a very attractive object for biotechnology.
